# Off Time Independently Affects Quality of Life in Advanced Parkinson's Disease (APD) Patients but Not in Non-APD Patients: Results from the Self-Reported Japanese Quality-of-Life Survey of Parkinson's Disease (JAQPAD) Study

**DOI:** 10.1155/2021/9917539

**Published:** 2021-10-12

**Authors:** Yuka Hayashi, Ryoko Nakagawa, Miwako Ishido, Yoko Yoshinaga, Jun Watanabe, Kanako Kurihara, Koichi Nagaki, Hiromu Ogura, Takayasu Mishima, Shinsuke Fujioka, Yoshio Tsuboi

**Affiliations:** ^1^Department of Neurology, Faculty of Medicine, Fukuoka University, 7-45-1 Nanakuma, Johnan-ku, Fukuoka 814-0180, Japan; ^2^Medical, AbbVie GK, 3-1-21 Shibaura, Minato-ku, Tokyo 108-0023, Japan

## Abstract

**Introduction:**

Parkinson's disease (PD) is characterized by a triad of motor symptoms and several nonmotor symptoms (NMS). Identifying the most appropriate treatment is essential for improving patient quality of life (QoL). However, it is still not known which PD symptoms more commonly affect patients with advanced PD (APD) versus non-APD. This study examined the factors that most affected the QoL of patients with APD (defined using the 5-2-1 criteria: ≥5 oral levodopa doses a day, off time ≥2 hours a day, or troublesome dyskinesia ≥1 hour a day) versus non-APD in a large Japanese population using the Japanese Quality-of-Life Survey of Parkinson's Disease (JAQPAD) study.

**Methods:**

Participants in this self-reported survey-based study included all members of the Japan Parkinson's Disease Association. Questionnaires assessing NMS and QoL (e.g., the 8-item PD Questionnaire [PDQ-8]) were included. Univariate and multivariate regression analyses were conducted to identify clinical factors impacting QoL using the PDQ-8 Summary Index (PDQ-8 SI).

**Results:**

Of the 3022 eligible patients, 864 were classified as having non-APD and 1599 as having APD. QoL as assessed by the PDQ-8 SI was notably worse in patients with APD versus non-APD (39.2 vs. 26.9, *p* < 0.0001). Although off time affected QoL only in patients with APD, PD duration and the NMS Questionnaire score significantly contributed to the QoL in both patients with APD and non-APD.

**Conclusions:**

This study identified the factors more commonly associated with worse QoL in patients with APD versus non-APD. Our findings offer new insights for providing optimal treatment and improving treatment satisfaction in patients with PD.

## 1. Introduction

Parkinson's disease (PD) is the fastest growing neurodegenerative disorder [1]. In 2016, PD affected approximately 6.1 million people globally and approximately 0.26 million people in Japan [1]. The prevalence of PD is expected to increase further owing to rapid aging of the Japanese population [2]. PD is defined by the triad of motor symptoms, namely, bradykinesia, rigidity, and tremor [3], as well as many nonmotor symptoms, such as neuropsychiatric symptoms, autonomic dysfunction, cognitive impairment, pain, and fatigue [4–6]. In general, PD is characterized by the progression of the disease from the early stages with minimal functional impairment to the advanced stage typified by limited mobility, severe motor deficits, risk of falls, and a significant impairment in the quality of life (QoL) [7, 8].

The effective management of PD at all disease stages requires a personalized approach. In particular, identifying the most appropriate treatment strategies for patients in the advanced stage of the disease is essential to stabilize symptoms and improve health-related QoL. However, the concept of “advanced PD” (APD) remains controversial and poorly defined, primarily owing to the absence of a diagnostic test, biomarker, or gold standard index clearly defining the advanced stage of PD [9–11]. Consequently, the World Health Organization's International Classification of Diseases coding system currently has no definition for APD [12], and this has resulted in the term being variably applied to patients in routine clinical practice. In addition, the international, multicenter National Parkinson's Foundation Quality Improvement Initiative study identified a subset of 187 patients with PD who had survived ≥20 years and were healthier than anticipated. The median rating for these patients on the Hoehn and Yahr (H&Y) scale was stage 3, with 75% reporting motor fluctuations; however, these patients had only mild cognitive impairment, and a majority (89%) continued to reside at home [13]. Such findings highlight the complexity of defining APD and suggest that the concept of APD may need to be individualized.

A robust definition of APD is required to enable identification of patients progressing toward APD. Early identification of patients with APD facilitates optimization of treatment regimens and timely referral to movement disorder specialists or specialized centers for improved quality of care and patient outcomes. Therefore, a consensus on indicators of suspected APD and eligibility for device-aided therapies (DATs) was recently reached by a panel of movement disorder specialists from 10 European countries. Using a Delphi-panel approach, suspected APD symptoms were defined as follows: ≥5 daily oral doses of levodopa, ≥2 hours a day of off time (i.e., time when PD symptoms return because the medication is not working optimally), or ≥1 hour a day with troublesome dyskinesia—the 5-2-1 diagnostic criteria [14]. Notably, a cross-sectional, international, observational study conducted in 2615 patients with PD in 18 countries (OBSERVE-PD) was the first to determine the proportion of patients with APD attending specialist PD clinics and demonstrate the clinical burden of APD [15]. Based on the physicians' global assessment of APD and the Delphi criteria, the study showed that patients with APD experienced a greater disease burden, in terms of motor and nonmotor symptoms, and a reduced QoL compared with patients with non-APD [15].

An accurate diagnosis of APD is essential because the emergence of new motor and nonmotor symptoms or the worsening of existing symptoms in the advanced stages of the disease is a major source of disability that significantly affects the QoL of patients and their caregivers [16]. However, studies evaluating the effect of APD on patient QoL are sparse, particularly in Asian countries [17]. Although PD was added to the list of intractable diseases in 1978 by the Japanese Ministry of Health, Labour, and Welfare [18], few qualitative studies have captured data on the overall effect of PD on daily life, globally and specifically in Japan [17, 18]. Therefore, the Japanese Quality-of-Life Survey of Parkinson's Disease (JAQPAD) study was conducted to investigate the effect of PD on the QoL of patients in Japan [19]. Results showed that off time and the severity of nonmotor symptoms were the major determinants of QoL in Japanese patients with PD. Here, we present the results from an analysis of a subgroup of patients from the JAQPAD study who met ≥1 of the 5-2-1 APD diagnostic criteria. The study was conducted to investigate the effect of APD on QoL and to identify the factors that most affected patient QoL.

## 2. Materials and Methods

### 2.1. Study Design

Full details of the study design have been reported previously [19]. In brief, this was an observational, cross-sectional, self-reported, survey-based study conducted in Japan. between August 14 and October 31, 2017. The study was conducted in accordance with the Declaration of Helsinki (1964), local laws and regulations, guidelines for Good Pharmacoepidemiology Practices in noninterventional studies, and the Japan Ethical Guidelines for Medical and Health Research Involving Human Subjects [20]. This study was approved by the external institutional review board of the nonprofit organization MINS (Tokyo, Japan; Approval #170517).

### 2.2. Study Methods

Study participants included invited members from the Japan Parkinson's Disease Association (JPDA). The JPDA is the largest association of patients with PD in Japan, with approximately 8000 members comprising patients with PD and their families. Patients were sent an informed consent form (ICF) and a study questionnaire by mail and were requested to complete the questionnaire themselves or with the support of their caregivers. Reply envelopes with completed ICFs and questionnaires, collected anonymously, were sent directly by patients to a contracted clinical research organization (CRO). A month after sending the initial questionnaires, the CRO sent reminder postcards to patients. A call center was set up to respond to inquiries from patients.

### 2.3. Patient Selection

Participants invited to participate in this study included all 8183 members of the JPDA, both patients and their caregivers. Adult patients with a self-reported confirmed diagnosis of idiopathic PD and who provided informed consent were included in the study. Patients whose medical information was missing or disqualifying (number of medication types or frequency of medication not provided, taking ≥14 different types of medications, or taking medication ≥9 times a day) were excluded from the study. The use of ≥14 types of medications and medication frequency ≥9 times a day were considered deviations from the permitted prescriptions for PD in Japan.

### 2.4. Study Outcomes and Assessments

Patients who met ≥1 of the 5-2-1 diagnostic criteria were classified as having APD. Patients who did not fulfill any of the 5-2-1 diagnostic criteria but who had a confirmed diagnosis of PD were classified as having non-APD.

The primary outcome of this study was to identify factors that most affected the QoL of patients with APD versus non-APD. The secondary outcome was to assess the effect of each of the three 5-2-1 diagnostic criteria, individually or in combination, on the QoL of patients with PD. The 8-item Parkinson's Disease Questionnaire Summary Index (PDQ-8 SI) scores, providing a single index of health status reflective of the 39-item Parkinson's Disease Questionnaire scores, were used to assess the QoL. The PDQ-8 SI rates eight specific items (activities of daily living, bodily discomfort, cognition, communication, emotional well-being, mobility, social support, and stigma) on a 5-point Likert scale [18]. Demographic and clinical data of all enrolled patients were collected, as described previously [19]. Permissions for use of the PDQ-8 (Oxford University), Nonmotor Symptom Questionnaire (NMSQ; International Parkinson and Movement Disorders Society), European Quality-of-Life 5-Dimension, 5-Level Version Questionnaire, Summary Index, and European Quality-of-Life–Visual Analogue Scale (Euro QoL Research Foundation) were obtained.

### 2.5. Statistical Analysis

Between-group comparisons were performed using independent *t* tests, Wilcoxon tests, or chi-square tests to analyze the factors that were statistically significantly different between APD and non-APD patients. To determine the factors influencing QoL in APD and non-APD patients, multiple regression analyses (univariate and multivariate) were conducted using generalized linear models to evaluate the relationship between the PDQ-8 SI score and duration of off time for patients with APD and non-APD, with age, sex, duration of PD, employment status, type of PD medication, NMSQ score, and the 9-item Wearing-Off Questionnaire (WOQ-9) score as independent variables. The final model included duration of off time as a forced-in covariate because it was considered the primary indicator of PD severity [21, 22].

## 3. Results

### 3.1. Baseline Demographics and Clinical Characteristics

**Figure 1:**
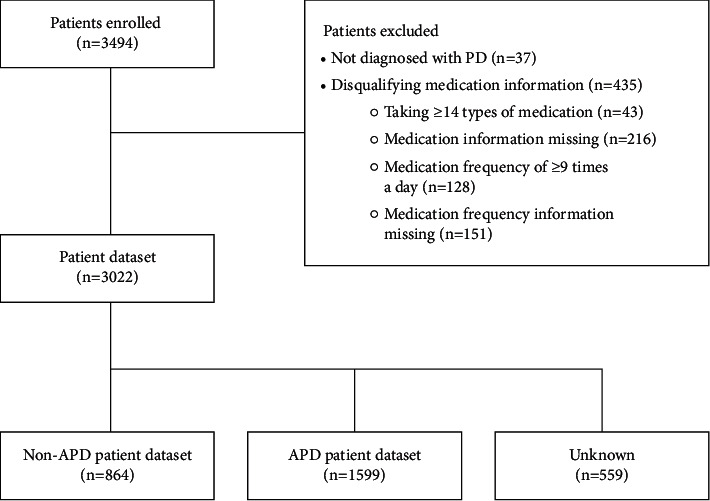
Patient disposition. This figure shows the disposition of patients who responded to the survey. The patient dataset comprises all patients who met the inclusion and exclusion criteria. Patients who met ≥1 of the 5-2-1 diagnostic criteria (≥5 oral levodopa doses a day, ≥2 hours of off time a day, troublesome dyskinesia ≥1 hour a day) were classified as having APD. Patients who did not meet any of the 5-2-1 diagnostic criteria were classified as having non-APD. Patients who could not be classified as having APD or non-APD because of missing or unknown data were classified as unknown. APD, advanced Parkinson's disease; PD, Parkinson's disease.

Overall, 3494 patients registered for this study; however, 472 patients were excluded because they self-reported not having a diagnosis of PD (*n* = 37) or inappropriate medication information was provided (*n* = 435). Of the 3022 remaining patients, 864 were classified as having non-APD and 1599 as having APD; 559 patients were classified as unknown because of missing or unknown data for “number of oral levodopa medications a day,” “duration of off time,” and “duration of troublesome dyskinesia” (Figure 1).

The baseline demographics and clinical characteristics of patients are shown in Table 1. The mean age of the patients was similar in the non-APD and APD groups (71.2 years and 70.5 years, respectively). Overall, the average age at PD diagnosis was higher in the non-APD group than in the APD group (63.6 vs. 58.6 years), and the mean duration of PD was greater in patients with APD (7.6 vs. 11.9 years). A majority of the patients in both groups were in H&Y stage 3, and there was a statistically significant difference between the H&Y stages in the non-APD and APD groups (*p* < 0.0001). All patients were receiving oral anti-PD medications. A higher percentage of patients with APD versus non-APD were receiving deep brain stimulation (10.3% vs. 6.9%). At baseline, wearing-off (i.e., reduction in medication effect and reemergence of PD symptoms over time) was detected by WOQ-9 assessment in 44.6% of patients in the non-APD group and 76.8% of patients in the APD group. QoL assessed by the PDQ-8 SI was notably worse in the APD group versus the non-APD group (39.2 vs. 26.9; Table 2). The mean (standard deviation) scores for each domain of the PDQ-8 scale were all higher in the APD group. Scores for nonmotor symptoms (measured by the NMSQ–Summary Index) and activities of daily living (measured by the Schwab and England Activities of Daily Living scale) and general QoL (measured by the European Quality-of-Life 5-Dimension, 5-Level Version Questionnaire Summary Index, and European Quality-of-Life–Visual Analogue Scale) were also worse in the APD group compared with the non-APD group. The scores for all domains of the NMSQ in patients with APD were also statistically significantly higher than in patients with non-APD (Table 3).

### 3.2. Factors Affecting Patients' QoL (Assessed by the PDQ-8 SI Score)

Results from the univariate analysis showed that off time, age, sex, duration of PD, employment status, type of PD medication, NMSQ score, and WOQ-9 score were individually predictive of changes in the PDQ-8 SI score. The variables sex, type of PD medication, and WOQ-9 score were excluded from the final multivariate model owing to a high correlation with the variable off time. The variable employment status was also excluded for APD patients.

Factors that contributed significantly to the QoL (PDQ-8 SI score) of patients with non-APD were duration of PD (*p*=0.0004), work status (*p*=0.0128), and NMSQ score (*p* < 0.0001). NMSQ score had the highest standardized correlation coefficient estimate (*r* = 0.564), followed by the duration of PD (*r* = 0.12; Table 4).

Similarly, factors that contributed significantly to the QoL of patients with APD were duration of off time (*p* < 0.0001), patient age (*p*=0.0212), duration of PD (*p* < 0.0001), and NMSQ score (*p* < 0.0001). The NMSQ score had the highest standardized correlation coefficient estimate (*r* = 0.557), followed by off time (*r* = 0.130; Table 5).

All three of the 5-2-1 criteria—the frequency of oral levodopa medication, duration of off time, and duration of troublesome dyskinesia—affected the PDQ-8 SI scores of all patients with PD (Table S1). Multivariate analysis showed that the duration of off time had the highest standardized correlation coefficient estimate (*r* = 0.189, *p* < 0.0001), followed by the duration of troublesome dyskinesia (*r* = 0.156, *p* < 0.0001) and the frequency of oral levodopa medications (*r* = 0.107, *p* < 0.0001).

### 3.3. Clinical Characteristics of Patients for Each 5-2-1 Criterion

The baseline demographics and clinical characteristics of patients meeting none to all three of the 5-2-1 criteria are shown in Table S2. Patients meeting only one of the 5-2-1 criteria had worse QoL scores (33.6, 35.5, and 39.3 for criteria 5, 2, and 1, respectively) than the non-APD population (26.9; Table 6). The demographics of the patients meeting versus not meeting each one of the 5-2-1 criteria in the APD population are shown in Table S3. Patients meeting either criterion 1 or criterion 2 had the worst QoL (PDQ-8 SI) scores (42.7 and 39.9, respectively), but, interestingly, those who met criterion 5 had almost no difference in the QoL score compared with those who did not (38.5 vs. 39.0, respectively; Table S4).

## 4. Discussion

Results from this observational, cross-sectional study provide a spectrum of indicators for assessing QoL in patients with APD. To the best of our knowledge, this study is the first to use the 5-2-1 diagnostic criteria to assess the effect of APD on patient QoL and determine the factors that most affect QoL in a Japanese population. Patient demographic data indicated that patients with APD represented a slightly younger population compared with the non-APD group, which is not consistent with the findings of the previous OBSERVE-PD study or with another study using the 5-2-1 diagnostic criteria in Spain [23]. These findings are also not in line with the results from a previous systematic review, which showed that older age is predictive of increasing disability [24]. On the other hand, a recent study has shown that older age at disease manifestation is associated with better outcomes in terms of disease progression to advanced stages [25]. Taken together, these results highlight the importance of personalized medicine and underscore the differences in aging trajectories among individuals, as reported in a recent seminal study [26]. In Japan, it is known that women are significantly more affected by PD [27, 28] and have a higher risk of wearing-off [29] than men, which is different from the observations in Europe and the US, where PD is known to affect men more commonly than women, with a similar rate of disease progression [30, 31] and severity between both sexes [30]. Interestingly, in the current study, women represented 59% of the APD group and 50% of the non-APD group. The recent seminal study also showed that clinical presentation, disease course, and health behavior differed between sexes [26], which is in line with our results. The longer duration of PD among patients with APD was in line with previous studies, as expected. Furthermore, similar to the results of a previous study [13], patients with APD experienced an earlier onset of disease, usually in the fifth decade, compared with the sixth decade for patients with non-APD. As expected, all patients were receiving oral anti-PD medications; however, not many patients with APD were receiving deep brain stimulation (APD, 10.3%; non-APD, 6.9%). This highlights the importance of identifying patients progressing to APD who may require advanced-stage treatment options, including DATs. Nonmotor symptoms, activities of daily living, and QoL scores were all worse among APD versus non-APD patients (*p* < 0.0001 for all). These findings suggest that the Delphi criteria for APD may be a useful assessment tool to aid patient classification and introduce the benefit of DAT for patients with APD.

Overall, PD duration and the NMSQ score significantly impacted QoL of patients in all stages of PD. It has been reported that the number of wearing-off symptoms increases with disease duration, negatively affecting patient QoL [21, 22]. Therefore, it is not surprising that patient QoL was adversely impacted by the duration of off time in our study [19]. Interestingly, the duration of off time impacted QoL for patients with APD but not for those with non-APD, possibly because of the shorter duration of off time for patients with non-APD (0.3 vs. 3.2 hours/day). However, our results show that longer disease duration was associated with a worsening of QoL in patients both with and without APD, which corroborates previous findings [32]. Notably, nonmotor symptoms, as assessed by the NMSQ, were shown to affect the QoL of patients with and without APD equally. These findings are in line with those from previous studies that have shown a negative correlation between nonmotor symptoms and QoL and highlighted that patients report nonmotor symptoms from the early to advanced stages of PD [33, 34]. Interestingly, results from previous studies on the effect of patients' age on QoL have been inconsistent [32], which has been attributed to cultural or ethnic differences across population groups. However, in our study, patient age had a significant effect on QoL in patients with APD. Such findings suggest that future studies are warranted to consider the extent to which demographic factors, such as age, may contribute to QoL.

A recent review of PD studies highlighted that “happiness,” which is a component of QoL, is an interesting and relevant outcome to consider in patients with PD [35]. Of note, approximately 70% of patients rated “happiness” as the most important parameter in life, thereby highlighting its use as a possible outcome measure in clinical trials in PD. However, further studies are required to assess meaningful endpoints for individual patients to decide treatment interventions.

Our study showed that meeting ≥1 of the 5-2-1 criteria actually worsened QoL (Tables 1 and 6), which indicates the usefulness of this index of criteria. Furthermore, factors that reduce QoL at each stage could be analyzed by categorizing patients into stages using the 5-2-1 criteria, which may be useful for treatment selection based on their condition. The study conducted in Spain also reported that QoL was worse in patients meeting ≥1 of the 5-2-1 criteria and suggested the usefulness of these criteria in identifying patients requiring optimization of PD treatment [23]. While the 5-2-1 criteria could be a helpful tool in identifying patients requiring a change in their clinical regimen, the definition and treatment of APD require further standardization. Recent studies have suggested that guidelines to assist clinicians and patients in choosing DATs for advanced therapy are required [36]. Recently, an international panel of movement disorder specialists developed the Making Informed Decisions to Aid Timely Management of Parkinson's Disease (MANAGE-PD) tool, an instrument designed to support healthcare providers in identifying patients with PD who are uncontrolled on oral medications [37]. This tool includes “unpredictable fluctuations of motor symptoms” and “limitations of activities of daily living” in addition to the 5-2-1 criteria for identifying whether or not patients are controlled on their current treatment regimen. In addition, the tool enables assessment of the frequency and severity of seven symptoms, including key motor symptoms, nonmotor symptoms, adverse events, and functional impact, which can help determine whether patients could benefit from further treatment optimization or if they should be considered for DAT.

This study has a few limitations. As the study included only those patients willing to complete a survey, there could have been a selection bias, which may limit the generalizability of the study. Moreover, all data, including those on PD diagnosis, were self-reported. The subjective nature of the questionnaire and its remote administration could also limit the validity of the results. The lack of information regarding medication use and the severity of motor symptoms assessed using clinical scales (e.g., Unified Parkinson's Disease Rating Scale-III and Scales for Outcomes in Parkinson's Disease—Motor) as well as the impact of cognitive impairment on reliable completion of the questionnaire without the presence of an interviewer must also be acknowledged. Lastly, distribution of patients by symptom severity could be negatively skewed because of the use of the JPDA. Therefore, the sample population may not be fully representative of the Japanese population. However, despite these limitations, the large sample size of the study allowed for an extensive investigation of the effect of PD on the QoL of patients in Japan.

## 5. Conclusion

This study helped identify factors that worsen QoL in patients with APD versus non-APD. Even in patients in the early stages of the disease whose off time duration had not affected QoL, nonmotor symptoms affected QoL significantly. These findings provide new insights for facilitating appropriate therapy and improving treatment satisfaction for patients with PD and suggest that the 5-2-1 criteria are a useful screening tool to identify patients with low QoL levels.

## Figures and Tables

**Table 1:** Baseline demographics and clinical characteristics.

	Patients		*p* value
	Non-APD *n* = 864	APD *n* = 1599	
*Patient demographics*			
Age, years, mean (SD)	71.2 (7.6)	70.5 (7.8)	0.0166
<65, *n* (%)	143 (16.6)	310 (19.4)	0.0835
≥65, *n* (%)	718 (83.1)	1284 (80.3)	
Sex, female, *n* (%)	433 (50.1)	946 (59.2)	<0.0001

*Medical history*			
Age at PD diagnosis, years, mean (SD)	63.6 (9.1)	58.6 (10.0)	<0.0001
Duration of PD, years, mean (SD)	7.6 (5.9)	11.9 (6.9)	<0.0001
H&Y stage, *n* (%)			
1	85 (9.8)	37 (2.3)	<0.0001
2	139 (16.1)	123 (7.7)	
3	301 (34.8)	682 (42.7)	
4	74 (8.6)	303 (18.9)	
5	16 (1.9)	73 (4.6)	

*Working status, n (%)*			
Full-time	42 (4.9)	44 (2.8)	<0.0001
Part-time	33 (3.8)	30 (1.9)	
Other (e.g., on leave from work)	26 (3.0)	51 (3.2)	
Student	2 (0.2)	0 (0.0)	
Housewife/househusband	272 (31.5)	560 (35.0)	
Seeking a job/unemployed	9 (1.0)	35 (2.2)	
Retired	338 (39.1)	533 (33.3)	
Other	131 (15.2)	324 (20.3)	

*PD treatment*			
Use of oral medication, *n* (%)	864 (100.0)	1599 (100.0)	
Number of oral medications a day, mean (SD)	3.2 (1.0)	4.7 (1.6)	<0.0001
Number of oral levodopa medications a day, mean (SD)	2.7 (1.0)	4.4 (1.7)	<0.0001
Number of oral medication types, mean (SD)	3.7 (2.2)	4.8 (2.6)	<0.0001
Use of device-aided therapy^*∗*^, *n* (%)			
DBS	60 (6.9)	164 (10.3)	0.0039
LCIG	5 (0.6)	17 (1.1)	0.2016

*PD symptoms*			
Duration of off time, hours/day, mean (SD)	0.3 (0.5)	3.2 (2.5)	<0.0001
Duration of troublesome dyskinesia, hours/day, mean (SD)	0.1 (0.2)	1.3 (2.0)	<0.0001
Presence of WO according to WOQ-9, *n* (%)	358 (44.6)	1037 (76.8)	<0.0001
NMSQ-TS, mean (SD)	12.8 (5.6)	16.2 (5.6)	<0.0001
SE-ADL, mean (SD)	74.4 (18.8)	61.8 (20.0)	<0.0001
PDQ-8 SI, mean (SD)	26.9 (18.3)	39.2 (20.5)	<0.0001
EQ-5D-5L-SI, mean (SD)	0.639 (0.200)	0.500 (0.201)	<0.0001
EQ-VAS, mean (SD)	66.3 (17.8)	56.6 (18.4)	<0.0001

Unknown/missing data are not listed. ^*∗*^Continuous subcutaneous apomorphine infusion is not approved in Japan. APD: advanced Parkinson's disease; DBS: deep brain stimulation; EQ-5D-5L-SI: European Quality-of-Life 5-Dimension, 5-Level Version Questionnaire Summary Index; EQ-VAS: European Quality-of-Life–Visual Analogue Scale; H&Y: Hoehn and Yahr; LCIG: levodopa-carbidopa intestinal gel; NMSQ-TS: Nonmotor Symptoms Questionnaire Total Score; PD: Parkinson's disease; PDQ-8 SI: 8-item Parkinson's Disease Questionnaire Summary Index; SD: standard deviation; SE-ADL: Schwab and England Activities of Daily Living; WO: wearing-off; WOQ-9: 9-item Wearing-Off Questionnaire.

**Table 2:** Summary of PDQ-8 domain scores.

	Patients, mean (SD)		*p* value
	Non-APD	APD	
*Domain*			
Difficulty getting around	40.7 (32.1)	55.6 (31.4)	<0.0001
Difficulty dressing	39.5 (32.4)	52.8 (30.8)	<0.0001
Felt depressed	28.9 (25.5)	41.7 (27.8)	<0.0001
Problems with close personal relationships	15.8 (22.9)	26.3 (28.5)	<0.0001
Problems with concentration	27.2 (26.7)	39.8 (29.2)	<0.0001
Felt unable to communicate properly	26.3 (27.0)	37.1 (29.9)	<0.0001
Painful muscle cramps or spasm	20.7 (25.3)	32.3 (30.8)	<0.0001
Felt embarrassed in public	17.0 (22.6)	29.0 (28.9)	<0.0001
Summary index	26.9 (18.3)	39.2 (20.5)	<0.0001

APD: advanced Parkinson's disease; PDQ-8: 8-item Parkinson's Disease Questionnaire; SD: standard deviation.

**Table 3:** Summary of NMSQ scores.

	Patients, mean (SD)		*p* value
	Non-APD	APD	
*Item*			
Digestive disorder	2.6 (1.6)	3.4 (1.7)	<0.0001
Urinary disorder	1.5 (0.7)	1.6 (0.6)	<0.0001
Memory disorder	1.8 (1.0)	2.1 (1.0)	<0.0001
Autonomic dysfunction	0.9 (0.7)	1.1 (0.7)	<0.0001
Sleep disorder	2.3 (1.4)	2.9 (1.4)	<0.0001
Perceptual disorder	0.6 (0.8)	0.9 (0.9)	<0.0001
Mood disorder	0.7 (0.9)	1.1 (0.9)	<0.0001
Sexual dysfunction	0.4 (0.7)	0.5 (0.8)	0.0114
Miscellaneous	2.2 (1.4)	2.8 (1.3)	<0.0001
Total score	12.8 (5.6)	16.2 (5.6)	<0.0001

APD: advanced Parkinson's disease; NMSQ: Nonmotor Symptoms Questionnaire; SD: standard deviation.

**Table 4:** Multiple regression analysis for PDQ-8 SI in patients with non-APD.

	Univariate model			Multivariate initial model			Multivariate final model backward elimination *p* < 0.05			
	CE	95% CI	*p* value	CE	95% CI	*p* value	CE	Standardized CE	95% CI	*p* value
Duration of off time	4.96	2.480, 7.440	<0.0001	0.928	−1.544, 3.400	0.4614	0.352	0.01	−1.973, 2.676	0.7665
Age	0.447	0.277, 0.617	<0.0001	0.152	−0.017, 0.321	0.077	0.159	0.066	−0.004, 0.321	0.0552
Sex (ref: female)	2.608	0.035, 5.180	0.047	0.229	−2.127, 2.585	0.8486				
Duration of PD	0.966	0.753, 1.179	<0.0001	0.393	0.175, 0.610	0.0004	0.376	0.12	0.170, 0.582	0.0004
Work status (ref: unemployed)	−7.753	−11.846, −3.661	0.0002	−4.849	−8.749, −0.949	0.0149	−4.822	−0.084	−8.616, −1.028	0.0128
Number of PD medication types	1.645	1.073, 2.217	<0.0001	−0.196	−0.790, 0.398	0.5168				
NMSQ-TS	1.956	1.751, 2.161	<0.0001	1.863	1.634, 2.091	<0.0001	1.823	0.564	1.608, 2.039	<0.0001
WOQ-9 (ref: absence)	2.934	0.328, 5.540	0.0274	−1.326	−3.883, 1.231	0.309				

APD: advanced Parkinson's disease; CE: coefficient estimate; CI: confidence interval; NMSQ-TS: Nonmotor Symptoms Questionnaire Total Score; PD: Parkinson's disease; PDQ-8 SI: 8-item Parkinson's Disease Questionnaire Summary Index; ref: reference; WOQ-9: 9-item Wearing-Off Questionnaire.

**Table 5:** Multiple regression analysis for PDQ-8 SI in patients with APD.

	Univariate model			Multivariate initial model			Multivariate final model backward elimination *p* < 0.05			
	CE	95% CI	*p* value	CE	95% CI	*p* value	CE	Standardized CE	95% CI	*p* value
Duration of off time	1.136	0.680, 1.593	<0.0001	1.015	0.579, 1.452	<0.0001	1.106	0.13	0.678, 1.533	<0.0001
Age	0.233	0.091, 0.374	0.0013	0.114	−0.028, 0.256	0.1141	0.154	0.06	0.023, 0.286	0.0212
Sex (ref: female)	3.399	1.137, 5.661	0.0033	1.059	−1.092, 3.210	0.3342				
Duration of PD	0.615	0.454, 0.777	<0.0001	0.389	0.219, 0.558	<0.0001	0.369	0.119	0.211, 0.527	<0.0001
Work status (ref: unemployed)	−4.746	−8.936, −0.556	0.0264	−1.481	−5.404, 2.442	0.4588				
Number of PD medication types	1.183	0.750, 1.615	<0.0001	0.071	−0.374, 0.517	0.7531				
NMSQ-TS	2.155	1.972, 2.339	<0.0001	2.028	1.834, 2.222	<0.0001	2.047	0.557	1.860, 2.235	<0.0001
WOQ-9 (ref: absence)	−2.095	−4.893, 0.703	0.1422	−1.718	−4.422, 0.985	0.2126				

APD: advanced Parkinson's disease; CE: coefficient estimate; CI: confidence interval; NMSQ-TS: Nonmotor Symptoms Questionnaire Total Score; PD: Parkinson's disease; PDQ-8 SI: 8-item Parkinson's Disease Questionnaire Summary Index; ref: reference; WOQ-9: 9-item Wearing-Off Questionnaire.

**Table 6:** PDQ-8 score assessed for patients meeting (+) or not meeting (−) each 5-2-1 criterion.

Item	*n* (%)	PDQ-8 SI, mean (SD)
5 (+) 2 (+) 1 (+)	158 (5.5)	44.4 (19.5)
5 (+) 2 (+) 1 (−)	208 (7.3)	37.8 (19.1)
5 (+) 2 (−) 1 (+)	47 (1.6)	36.5 (17.7)
5 (−) 2 (+) 1 (+)	188 (6.6)	43.7 (22.2)
5 (+) 2 (−) 1 (−)	163 (5.7)	33.6 (19.1)
5 (−) 2 (+) 1 (−)	332 (11.6)	35.5 (20.1)
5 (−) 2 (−) 1 (+)	92 (3.2)	39.3 (19.3)
5 (−) 2 (−) 1 (−)	829 (29.1)	26.9 (18.3)

1: troublesome dyskinesia ≥1 hour a day; 2: ≥2 hours of off time a day; 5: ≥5 oral levodopa doses a day; PDQ-8 SI: 8-item Parkinson's Disease Questionnaire Summary Index; SD: standard deviation.

## Data Availability

The data generated and/or analyzed during the current study that were used to support the findings of the study were supplied by AbbVie under license and therefore cannot be made freely available. Requests for access to these data should be made to the corresponding author.

## Supplementary Materials

Supplementary Materials Table S1: Multiple regression analysis for PDQ-8 SI. Table S2: Baseline demographics and clinical characteristics of patients meeting (+) or not meeting (−) all of the 5-2-1 criteria. Table S3: Baseline demographics of patients with APD meeting (+) or not meeting (−) each of the 5-2-1 criteria. Table S4: PDQ-8 SI score assessed in patients with APD meeting (+) or not meeting (−) each of the 5-2-1 criteria.

## References

[1] GBD 2016 Parkinson’s Disease Collaborators (2018). Global, regional, and national burden of Parkinson’s disease, 1990–2016: a systematic analysis for the global burden of disease study 2016. *The Lancet Neurology*.

[2] Osaki Y., Morita Y., Kuwahara T., Miyano I., Doi Y. (2011). Prevalence of Parkinson’s disease and atypical parkinsonian syndromes in a rural Japanese district. *Acta Neurologica Scandinavica*.

[3] Hughes A. J., Daniel S. E., Kilford L., Lees A. J. (1992). Accuracy of clinical diagnosis of idiopathic Parkinson’s disease: a clinico-pathological study of 100 cases. *Journal of Neurology, Neurosurgery & Psychiatry*.

[4] Titova N., Martinez-Martin P., Katunina E., Chaudhuri K. R. (2017). Advanced Parkinson’s or “complex phase” Parkinson’s disease? re-evaluation is needed. *Journal of Neural Transmission*.

[5] Carrarini C., Russo M., Dono F. (2019). A stage-based approach to therapy in Parkinson’s disease. *Biomolecules*.

[6] Chaudhuri K. R., Healy D. G., Schapira A. H., National Institute for Clinical Excellence (2006). Non-motor symptoms of Parkinson’s disease: diagnosis and management. *The Lancet Neurology*.

[7] Varanese S., Birnbaum Z., Rossi R., Rocco A. D. (2010). Treatment of advanced Parkinson’s disease. *Parkinson’s Disease*.

[8] Poewe W., Mahlknecht P. (2009). The clinical progression of Parkinson’s disease. *Parkinsonism & Related Disorders*.

[9] Riedel O., Klotsche J., Wittchen H.-U., GEPAD Study Group (2014). Motor impairment, depression, dementia: which forms the impression of disease severity in Parkinson’s disease?. *Parkinsonism and Related Disorders*.

[10] Martínez-Martín P., Rodríguez-Blázquez C., Alvarez M. (2015). Parkinson’s disease severity levels and MDS-unified Parkinson’s disease rating scale. *Parkinsonism and Related Disorders*.

[11] Luquin M. R., Kulisevsky J., Martinez-Martin P., Mir P., Tolosa E. S. (2017). Consensus on the definition of advanced Parkinson’s disease: a neurologists-based Delphi study (CEPA study). *Parkinson’s Disease*.

[12] World Health Organization (2021). *International Statistical Classification of Diseases and Related Health Problems 10th Revision (ICD-10) Version 2019, ICD Revision Topic Advisory Groups*.

[13] Hassan A., Wu S. S., Schmidt P. (2015). The profile of long-term Parkinson’s disease survivors with 20 years of disease duration and beyond. *Journal of Parkinson’s Disease*.

[14] Antonini A., Stoessl A. J., Kleinman L. S. (2018). Developing consensus among movement disorder specialists on clinical indicators for identification and management of advanced Parkinson’s disease: a multi-country Delphi-panel approach. *Current Medical Research and Opinion*.

[15] Fasano A., Fung V. S. C., Lopiano L. (2019). Characterizing advanced Parkinson’s disease: OBSERVE-PD observational study results of 2615 patients. *BMC Neurology*.

[16] Martínez-Martin P., Stocchi F., Reichmann H. (2014). Quality of life in Parkinson’s disease—patient, clinical and research perspectives. *European Neurological Review*.

[17] Abbas M. M., Xu Z., Tan L. C. S. (2018). Epidemiology of Parkinson’s disease-east versus west. *Movement Disorders Clinical Practice*.

[18] Morioka S., Sakata K., Yoshida S. (2002). Incidence of Parkinson disease in Wakayama, Japan. *Journal of Epidemiology*.

[19] Kurihara K., Nakagawa R., Ishido M. (2020). Impact of motor and nonmotor symptoms in Parkinson disease for the quality of life: the Japanese Quality-of-Life Survey of Parkinson Disease (JAQPAD) study. *Journal of the Neurological Sciences*.

[20] International Society for Pharmacoepidemiology Guidelines for good pharmacoepidemiology practices (GPP). https://www.pharmacoepi.org/resources/policies/guidelines-08027/.

[21] Stocchi F., Antonini A., Barone P. (2014). Early DEtection of wEaring off in Parkinson disease: the DEEP study. *Parkinsonism and Related Disorders*.

[22] Rodríguez-Violante M., Ospina-García N., Dávila-Avila N. M., Cruz-Fino D., Cruz-Landero A. d. l., Cervantes-Arriaga A. (2018). Motor and non-motor wearing-off and its impact in the quality of life of patients with Parkinson’s disease. *Arquivos de Neuro-psiquiatria*.

[23] Santos-Garcia D., de Deus Fonticoba T., Suarez Castro E., Aneiros Diaz A., McAfee D. (2020). 5-2-1 criteria: a simple screening tool for identifying advanced PD patients who need an optimization of Parkinson’s treatment. *Parkinson’s Disease*.

[24] Marras C., Rochon P., Lang A. E. (2002). Predicting motor decline and disability in Parkinson disease: a systematic review. *Archives of Neurology*.

[25] Mahajan A., Chirra M., Dwivedi A. K. (2020). Skin cancer may delay onset but not progression of Parkinson’s disease: a nested case-control study. *Frontiers in Neurology*.

[26] Bloem B. R., Okun M. S., Klein C. (2021). Parkinson’s disease. *The Lancet*.

[27] Kimura H., Kurimura M., Wada M. (2002). Female preponderance of Parkinson’s disease in Japan. *Neuroepidemiology*.

[28] Yamawaki M., Kusumi M., Kowa H., Nakashima K. (2009). Changes in prevalence and incidence of Parkinson’s disease in Japan during a quarter of a century. *Neuroepidemiology*.

[29] Ouma S., Fukae J., Fujioka S. (2017). The risk factors for the wearing-off phenomenon in Parkinson’s disease in Japan: a cross-sectional, multicenter study. *Internal Medicine (Tokyo, Japan)*.

[30] Bower J. H., Maraganore D. M., McDonnell S. K., Rocca W. A. (2000). Influence of strict, intermediate, and broad diagnostic criteria on the age- and sex-specific incidence of Parkinson’s disease. *Movement Disorders*.

[31] Baldereschi M., Di Carlo A., Rocca W. A. (2000). Parkinson’s disease and parkinsonism in a longitudinal study: two-fold higher incidence in men. ILSA working group. Italian longitudinal study on aging. *Neurology*.

[32] Antonini A., Robieson W. Z., Bergmann L., Yegin A., Poewe W. (2018). Age/disease duration influence on activities of daily living and quality of life after levodopa-carbidopa intestinal gel in Parkinson’s disease. *Neurodegenerative Disease Management*.

[33] Khoo T. K., Yarnall A. J., Duncan G. W. (2013). The spectrum of nonmotor symptoms in early Parkinson disease. *Neurology*.

[34] Barone P., Antonini A., Colosimo C. (2009). The PRIAMO study: a multicenter assessment of nonmotor symptoms and their impact on quality of life in Parkinson’s disease. *Movement Disorders*.

[35] Cools C. I., de Vries N. M., Bloem B. R. (2020). Happiness: a novel outcome in Parkinson studies?. *Journal of Parkinson’s Disease*.

[36] Marsili L., Bologna M., Miyasaki J. M., Colosimo C. (2021). Device-aided therapies for advanced Parkinson disease: insights from an international survey. *Neurological Sciences*.

[37] AbbVie Inc. Tool for Making Informed Decisions to Aid Timely Management of Parkinson's Disease (MANAGE-PD). https://www.managepd.com/.

[38] Tsuboi Y., Nakagawa R., Ishido M. (2019). The quality of life burden in advanced Parkinson’s disease applying “5-2-1” diagnosing criteria: subgroup analyses of the JAQPAD (Japanese QOL survey of Parkinson’s disease) study. *European Journal of Neurology*.

[39] Tsuboi Y., Ishido M., Watanabe J. (2020). Off-time independently affects to QOL in advanced Parkinson’s disease (APD) patients, but not in non-APD patients; an explanatory analysis of the JAQPAD study. *European Journal of Neurology*.

