# Supplementary material for: Off Time Independently Affects Quality of Life in Advanced Parkinson's Disease (APD) Patients but Not in Non-APD Patients: Results from the Self-Reported Japanese Quality-of-Life Survey of Parkinson's Disease (JAQPAD) Study
Source: Parkinsons Dis. 2021 Oct 12;2021:9917539. doi: 10.1155/2021/9917539 (PMC8526216; doi:10.1155/2021/9917539)
Supplement: Supplementary Materials — Table S1: Multiple regression analysis for PDQ-8 SI. Table S2: Baseline demographics and clinical characteristics of patients meeting (+) or not meeting (−) all of the 5-2-1 criteria. Table S3: Baseline demographics of patients with APD meeting (+) or not meeting (−) each of the 5-2-1 criteria. Table S4: PDQ-8 SI score assessed in patients with APD meeting (+) or not meeting (−) each of the 5-2-1 criteria. [file 9917539.f1.docx]

## Supplementary Material

**Table S1:** Multiple regression analysis for PDQ-8 SI.

| **Factor** | **Value of the factor,**  **mean (SD)** | **PDQ-8 SI, mean (SD)** | **Univariate model** | | |  | **Multivariate initial model** | | |
| --- | --- | --- | --- | --- | --- | --- | --- | --- | --- |
|  |  |  | **CE** | **95% CI** | ***p* value^a^** | **CE** | **Standardized CE** | **95% CI** | ***p* value*** |
| Frequency of oral levodopa medications | 3.7 (1.7) | 34.32 (20.44) | 2.015 | 1.518, 2.511 | <0.0001 | 1.308 | 0.107 | 0.784, 1.831 | <0.0001 |
| Duration of off time | 1.9 (2.4) | 33.83 (33.83) | 2.342 | 2.015, 2.669 | <0.0001 | 1.624 | 0.189 | 1.245, 2.003 | <0.0001 |
| Duration of troublesome dyskinesia | 0.74 (1.6) | 33.99 (33.99) | 2.827 | 2.355, 3.299 | <0.0001 | 1.963 | 0.156 | 1.420, 2.505 | <0.0001 |

**p* value for the factor.

CE: coefficient estimate; CI: confidence interval; PDQ-8 SI: 8-item Parkinson’s Disease Questionnaire Summary Index; SD: standard deviation.

**Table S2:** Baseline demographics and clinical characteristics of patients meeting (+) or not meeting (–) all of the 5-2-1 criteria.

|  | **5 (+) 2 (+) 1 (+)**  **n=158** | **5 (+) 2 (+) 1 (–)**  **n=208** | **5 (+) 2 (–) 1 (+)**  **n=47** | **5 (–) 2 (+) 1 (+)**  **n=188** | **5 (+) 2 (–) 1 (–)**  **n=163** | **5 (–) 2 (+) 1 (–)**  **n=332** | **5 (–) 2 (–) 1 (+)**  **n=92** | **5 (–) 2 (–) 1 (–)**  **n=829** |
| --- | --- | --- | --- | --- | --- | --- | --- | --- |
| Patient demographics | | | | | | | | |
| Age, years, mean (SD) | 69.2 (8.5) | 69.0 (7.8) | 71.0 (7.0) | 70.0 (8.0) | 70.4 (7.6) | 70.2 (7.7) | 72.5 (7.9) | 71.2 (7.6) |
| <65, n (%) | 46 (27.1) | 49 (22.6) | 8 (16.7) | 37 (18.9) | 30 (17.5) | 74 (21.0) | 13 (14.1) | 143 (16.6) |
| ≥65, n (%) | 123 (72.4) | 168 (77.4) | 40 (83.3) | 158 (80.6) | 141 (82.5) | 277 (78.5) | 79 (85.9) | 718 (83.1) |
| Sex, female, n (%) | 115 (67.6) | 124 (57.1) | 29 (60.4) | 125 (63.8) | 93 (54.4) | 179 (50.7) | 61 (66.3) | 433 (50.1) |
| Medical history | | | | | | | | |
| Age at PD diagnosis, years, mean (SD) | 54.4 (9.6) | 56.9 (9.0) | 57.3 (9.9) | 57.4 (10.5) | 58.8 (10.2) | 60.5 (8.8) | 61.5 (11.1) | 63.6 (9.1) |
| Duration of PD, years, mean (SD) | 14.8 (6.3) | 12.1 (6.5) | 13.7 (5.4) | 12.8 (7.6) | 11.6 (6.8) | 9.7 (6.1) | 11.0 (6.9) | 7.6 (5.9) |
| H&Y stage, n (%) |  |  |  |  |  |  |  |  |
| 1 | 0 (0.0) | 0 (0.0) | 0 (0.0) | 3 (1.5) | 7 (4.1) | 17 (4.8) | 3 (3.3) | 85 (9.8) |
| 2 | 9 (5.3) | 11 (5.1) | 5 (10.4) | 11 (5.6) | 15 (8.8) | 27 (7.6) | 10 (10.9) | 139 (16.1) |
| 3 | 59 (34.7) | 103 (47.5) | 27 (56.3) | 85 (43.4) | 84 (49.1) | 164 (46.5) | 32 (34.8) | 301 (34.8) |
| 4 | 64 (37.6) | 48 (22.1) | 7 (14.6) | 38 (19.4) | 33 (19.3) | 52 (14.7) | 13 (14.1) | 74 (8.6) |
| 5 | 13 (7.6) | 13 (6.0) | 1 (2.1) | 12 (6.1) | 6 (3.5) | 18 (5.1) | 1 (1.1) | 16 (1.9) |
| Working status, n (%) | | | | | | | | |
| Full-time | 2 (1.2) | 5 (2.3) | 3 (6.3) | 4 (2.0) | 5 (2.9) | 12 (3.4) | 2 (2.2) | 42 (4.9) |
| Part-time | 2 (1.2) | 4 (1.8) | 0 (0.0) | 4 (2.0) | 5 (2.9) | 7 (2.0) | 1 (1.1) | 33 (3.8) |
| Others (e.g., on leave from work) | 7 (4.1) | 8 (3.7) | 0 (0.0) | 3 (1.5) | 5 (2.9) | 15 (4.2) | 2 (2.2) | 26 (3.0) |
| Student | 0 (0.0) | 0 (0.0) | 0 (0.0) | 0 (0.0) | 0 (0.0) | 0 (0.0) | 0 (0.0) | 2 (0.2) |
| Housewife/househusband | 66 (38.8) | 84 (38.7) | 18 (37.5) | 77 (39.3) | 49 (28.7) | 113 (32.0) | 34 (37.0) | 272 (31.5) |
| Seeking a job/unemployed | 3 (1.8) | 4 (1.8) | 3 (6.3) | 4 (2.0) | 5 (2.9) | 2 (0.6) | 7 (7.6) | 9 (1.0) |
| Retired | 41 (24.1) | 68 (31.3) | 10 (20.8) | 57 (29.1) | 71 (41.5) | 144 (40.8) | 30 (32.6) | 338 (39.1) |
| Other | 47 (27.6) | 43 (19.8) | 13 (27.1) | 44 (22.4) | 29 (17.0) | 12 (3.4) | 14 (15.2) | 131 (15.2) |
| PD treatment | | | | | | | | |
| Use of oral medication, n (%) | 170 (100.0) | 217 (100.0) | 48 (100.0) | 196 (100.0) | 171 (100.0) | 353 (100.0) | 92 (100.0) | 864 (100.0) |
| Number of oral medications a day, mean (SD) | 6.1 (1.2) | 5.9 (1.2) | 6.1 (1.4) | 3.9 (1.2) | 5.6 (1.3) | 3.7 (1.1) | 3.5 (1.0) | 3.2 (1.0) |
| Number of oral levodopa medications a day, mean (SD) | 5.8 (1.0) | 5.8 (0.9) | 5.9 (1.1) | 3.1 (1.1) | 5.6 (0.8) | 3.1 (0.9) | 2.9 (1.1) | 2.7 (1.0) |
| Number of oral medication types, mean (SD) | 5.6 (2.8) | 5.0 (2.5) | 5.0 (2.2) | 5.2 (2.6) | 4.8 (2.7) | 4.2 (2.2) | 4.8 (2.8) | 3.7 (2.2) |
| Use of device-aided therapy, n (%) |  |  |  |  |  |  |  |  |
| DBS | 20 (11.8) | 31 (14.3) | 2 (4.2) | 24 (12.2) | 26 (15.2) | 18 (5.1) | 12 (13.0) | 60 (6.9) |
| LCIG | 0 (0.0) | 3 (1.4) | 0 (0.0) | 1 (0.5) | 0 (0.0) | 4 (1.1) | 1 (1.1) | 5 (0.6) |
| PD symptoms | | | | | | | | |
| Duration of off time, hours/day, mean (SD) | 4.25 (2.49) | 3.78 (2.30) | 1.04 (0.54) | 3.83 (2.01) | 0.72 (0.61) | 3.96 (2.62) | 0.72 (0.64) | 0.30 (0.51) |
| Duration of troublesome dyskinesia, hours/day, mean (SD) | 2.95 (2.40) | 0.17 (0.28) | 1.86 (0.84) | 2.74 (1.97) | 0.15 (0.26) | 0.10 (0.24) | 2.91 (3.07) | 0.06 (0.18) |
| PDQ-8 SI, mean (SD) | 44.37 (19.53) | 37.82 (19.08) | 36.50 (17.68) | 43.71 (22.22) | 33.56 (19.11) | 35.50 (20.09) | 39.28 (19.25) | 26.94 (18.33) |

Unknown/missing data are not listed.

1: troublesome dyskinesia ≥1 hour a day; 2: ≥2 hours of off time a day; 5: ≥5 oral levodopa doses a day; DBS: deep brain stimulation; H&Y: Hoehn and Yahr; LCIG: levodopa-carbidopa intestinal gel; PD: Parkinson’s disease; PDQ-8 SI: 8-item Parkinson’s Disease Questionnaire Summary Index; SD: standard deviation.

**Table S3:** Baseline demographics of patients with APD meeting (+) or not meeting (–) each of the 5-2-1 criteria.

|  | **5 (+)**  **n=695** | **5 (–)**  **n=684** | ***p* value** | **2 (+)**  **n=1156** | **2 (–)**  **n=357** | ***p* value** | **1 (+)**  **n=643** | **1 (–)**  **n=848** | ***p* value** |
| --- | --- | --- | --- | --- | --- | --- | --- | --- | --- |
| Patient demographics |  |  |  |  |  |  |  |  |  |
| Age, years,  mean (SD) | 69.8 (7.8) | 70.4 (7.8) | 0.1110 | 70.1 (7.9) | 71.4 (7.7) | 0.0068 | 70.8 (8.1) | 70.2 (7.7) | 0.1054 |
| <65, n (%) | 149 (21.4) | 133 (19.4) | 0.3731 | 239 (20.7) | 56 (15.7) | 0.0346 | 123 (19.1) | 166 (19.6) | 0.8345 |
| ≥65, n (%) | 545 (78.4) | 548 (80.1) |  | 912 (78.9) | 301 (84.3) |  | 518 (80.6) | 680 (80.2) |  |
| Sex, female, n (%) | 417 (60.0) | 392 (57.3) | 0.3100 | 682 (59.0) | 215 (60.2) | 0.6799 | 420 (65.3) | 454 (53.5) | <0.0001 |
| Medical history | | | | | | | | | |
| Age at PD diagnosis, years, mean (SD) | 56.9 (9.6) | 59.7 (9.9) | <0.0001 | 58.3 (9.7) | 59.6 (10.5) | 0.0372 | 57.8 (10.8) | 59.3 (9.4) | 0.0053 |
| Duration of PD, years, mean (SD) | 12.9 (6.5) | 10.8 (7.0) | <0.0001 | 11.8 (7.0) | 11.8 (6.7) | 0.9847 | 13.0 (7.1) | 10.9 (6.6) | <0.0001 |
| H&Y stage, n (%) |  |  |  |  |  |  |  |  |  |
| 1 | 8 (1.2) | 23 (3.4) | 0.0006 | 25 (2.2) | 12 (3.4) | 0.0783 | 9 (1.4) | 27 (3.2) | 0.0079 |
| 2 | 45 (6.5) | 53 (7.7) |  | 81 (7.0) | 33 (9.2) |  | 47 (7.3) | 67 (7.9) |  |
| 3 | 310 (44.6) | 301 (44.0) |  | 494 (42.7) | 157 (44.0) |  | 247 (38.4) | 390 (46.0) |  |
| 4 | 170 (24.5) | 108 (15.8) |  | 225 (19.5) | 61 (17.1) |  | 142 (22.1) | 144 (17.0) |  |
| 5 | 37 (5.3) | 34 (5.0) |  | 59 (5.1) | 9 (2.5) |  | 29 (4.5) | 40 (4.7) |  |
| Working status, n (%) | | | | | | | | | |
| Full-time | 18 (2.6) | 19 (2.8) | 0.9356 | 30 (2.6) | 12 (3.4) | 0.0031 | 16 (2.5) | 24 (2.8) | <0.0001 |
| Part-time | 11 (1.6) | 13 (1.9) |  | 22 (1.9) | 8 (2.2) |  | 12 (1.9) | 16 (1.9) |  |
| Others (e.g., on leave from work) | 22 (3.2) | 21 (3.1) |  | 42 (3.6) | 7 (2.0) |  | 18 (2.8) | 30 (3.5) |  |
| Student | 0 (0.0) | 0 (0.0) |  | 0 (0.0) | 0 (0.0) |  | 0 (0.0) | 0 (0.0) |  |
| Housewife/  househusband | 248 (35.7) | 243 (35.5) |  | 409 (35.4) | 121 (33.9) |  | 251 (39.0) | 269 (31.7) |  |
| Seeking a job/unemployed | 17 (2.4) | 14 (2.0) |  | 17 (1.5) | 18 (5.0) |  | 19 (3.0) | 13 (1.5) |  |
| Retired | 224 (32.2) | 245 (35.8) |  | 377 (32.6) | 120 (33.6) |  | 167 (26.0) | 330 (38.9) |  |
| Others | 146 (21.0) | 121 (17.7) |  | 245 (21.2) | 66 (18.5) |  | 149 (23.2) | 158 (18.6) |  |
| PD treatment | | | | | | | | | |
| Use of oral medication, n (%) | 695 (100.0) | 684 (100.0) |  | 1156 (100.0) | 357 (100.0) |  | 643 (100.0) | 848 (100.0) |  |
| Number of oral medications a day, mean (SD) | 5.9 (1.3) | 3.7 (1.1) | <0.0001 | 4.6 (1.6) | 4.9 (1.6) | 0.0019 | 4.7 (1.7) | 4.7 (1.6) | 0.5106 |
| Number of oral levodopa medications a day, mean (SD) | 5.8 (0.9) | 3.0 (1.0) | <0.0001 | 4.2 (1.7) | 4.9 (1.6) | <0.0001 | 4.2 (1.8) | 4.5 (1.6) | 0.0079 |
| Number of oral medication types, mean (SD) | 5.1 (2.7) | 4.6 (2.5) | 0.0003 | 4.8 (2.5) | 4.8 (2.6) | 0.7403 | 5.1 (2.7) | 4.5 (2.5) | <0.0001 |
| Use of device-aided therapy |  |  |  |  |  |  |  |  |  |
| DBS, n (%) | 95 (13.7) | 57 (8.3) | 0.0008 | 109 (9.4) | 43 (12.0) | 0.1709 | 67 (10.4) | 84 (9.9) | 0.6814 |
| LCIG, n (%) | 5 (0.7) | 7 (1.0) | 0.5727 | 13 (1.1) | 2 (0.6) | 0.3515 | 5 (0.8) | 11 (1.3) | 0.3535 |
| PD symptoms | | | | | | | | | |
| Duration of off time, hours/day, mean (SD) | 2.87 (2.53) | 3.52 (2.58) | <0.0001 | 3.94 (2.40) | 0.77 (0.63) | <0.0001 | 3.07 (2.40) | 3.20 (2.52) | 0.3236 |
| Duration of troublesome dyskinesia, hours/day, mean (SD) | 1.08 (1.81) | 1.35 (2.11) | 0.0127 | 1.22 (1.90) | 1.43 (2.30) | 0.1209 | 2.85 (2.33) | 0.14 (0.26) | <0.0001 |
| Presence of WO according to WOQ-9, n (%) | 564 (81.2) | 473 (72.1) | 0.0001 | 745 (77.8) | 240 (75.2) | 0.3963 | 399 (77.9) | 576 (77.1) | 0.4703 |
| NMSQ-SI, mean (SD) | 16.5 (5.5) | 15.8 (5.7) | 0.0391 | 16.1 (5.7) | 15.7 (5.3) | 0.3457 | 17.2 (5.6) | 15.4 (5.5) | <0.0001 |
| SE-ADL, mean (SD) | 60.8 (19.7) | 62.8 (20.3) | 0.0650 | 60.7 (20.2) | 65.8 (18.7) | <0.0001 | 59.0 (20.0) | 63.9 (19.8) | <0.0001 |
| PDQ-Carer SI, mean (SD) | 40.5 (23.8) | 40.7 (24.0) | 0.9364 | 42.1 (24.2) | 36.9 (23.2) | 0.0068 | 42.7 (24.4) | 40.1 (23.6) | 0.1138 |
| EQ-5D-5L-SI, mean (SD) | 0.50 (0.19) | 0.51 (0.20) | 0.2305 | 0.49 (0.20) | 0.53 (0.20) | 0.0077 | 0.48 (0.20) | 0.52 (0.20) | <0.0001 |
| EQ-VAS, mean (SD) | 57.1 (18.9) | 56.3 (18.1) | 0.4735 | 56.1 (18.1) | 59.1 (18.4) | 0.0078 | 54.9 (18.2) | 58.2 (18.3) | 0.0008 |

Unknown/missing data are not listed.

1: troublesome dyskinesia ≥1 hour a day; 2: ≥2 hours of off time a day; 5: ≥5 oral levodopa doses a day; APD: advanced Parkinson’s disease; DBS: deep brain stimulation; EQ-5D-5L-SI: European Quality-of-Life 5-Dimension, 5-Level Version Questionnaire Summary Index; EQ-VAS: European Quality-of-Life–Visual Analogue Scale; H&Y: Hoehn and Yahr; LCIG: levodopa-carbidopa intestinal gel; NMSQ-SI: Nonmotor Symptoms Questionnaire Summary Index; PD: Parkinson’s disease; PDQ-Carer SI: Parkinson’s Disease Questionnaire–Carer Summary Index; SD: standard deviation; SE-ADL: Schwab and England Activities of Daily Living; WO: wearing-off; WOQ-9: 9-item Wearing-Off Questionnaire.

**Table S4:** PDQ-8 SI score assessed in patients with APD meeting (+) or not meeting (–) each of the 5-2-1 criteria.

| **Item** | **N** | **PDQ-8 SI, mean (SD)** | ***p* value** |
| --- | --- | --- | --- |
| 5 (+) | 654 | 38.5 (19.5) | 0.64 |
| 5 (–) | 652 | 39.0 (19.1) |  |
| 2 (+) | 1091 | 39.9 (20.5) | 0.0021 |
| 2 (–) | 345 | 36.0 (19.6) |  |
| 1 (+) | 611 | 42.7 (20.8) | <0.0001 |
| 1 (–) | 800 | 36.5 (19.9) |  |

1: troublesome dyskinesia ≥1 hour a day; 2: ≥2 hours of off time a day; 5: ≥5 oral levodopa doses a day; APD: advanced Parkinson’s disease; PDQ-8 SI: 8-item Parkinson’s Disease Questionnaire Summary Index; SD: standard deviation.
